# Global rainfall erosivity database (GloREDa) and monthly R-factor data at 1 km spatial resolution

**DOI:** 10.1016/j.dib.2023.109482

**Published:** 2023-08-09

**Authors:** Panos Panagos, Tomislav Hengl, Ichsani Wheeler, Pawel Marcinkowski, Montfort Bagalwa Rukeza, Bofu Yu, Jae E. Yang, Chiyuan Miao, Nabansu Chattopadhyay, Seyed Hamidreza Sadeghi, Yoav Levi, Gunay Erpul, Christian Birkel, Natalia Hoyos, Paulo Tarso S. Oliveira, Carlos A. Bonilla, Werner Nel, Hassan Al Dashti, Nejc Bezak, Kristof Van Oost, Sašo Petan, Ayele Almaw Fenta, Nigussie Haregeweyn, Mario Pérez-Bidegain, Leonidas Liakos, Cristiano Ballabio, Pasquale Borrelli

**Affiliations:** a European Commission, Joint Research Centre (JRC), Ispra, 21027, Italy; b OpenGeoHub Foundation, 6708PW Wageningen, Netherlands; c Warsaw University of Life Sciences, Warsaw, Poland; d Observatoire Volcanologique de Goma, DR Congo; e Earth and Life Institute, UCLouvain, Belgium; f School of Engineering and Built Environment, Griffith University, Nathan, Australia; g Kangwon National University, Chuncheon-si, Gangwon-do, South Korea; h State Key Laboratory of Earth Surface Processes and Resource Ecology, Faculty of Geographical Science, Beijing Normal University, Beijing 100875, China; i World Bank, Washington, DC 20433, USA; j Faculty of Natural Resources, Tarbiat Modares University (TMU), Mazandaran Province, Iran; k Israel Meteorological Service, Bet Dagan, Israel; l Faculty of Agriculture - Soil Science Departement, Ankara University, Ankara, Turkey; m University of Costa Rica, San Jose, Costa Rica; n Universidad del Norte, Barranquilla, Colombia; o Federal University of Mato Grosso do Sul, Campo Grande, Brazil; p Hermiston Agricultural Research and Extension Center, Oregon State University, Hermiston, OR, USA; q Department of Geography and Environmental Science, University of Fort Hare, Alice, South Africa; r Department of Meteorology - Directorate General of Civil Aviation, State of Kuwait; s University of Ljubljana, Faculty of Civil and Geodetic Engineering, Ljubljana, Slovenia; t Slovenian Environment Agency, Meteorology, Hydrology and Oceanography Office, Ljubljana, Slovenia; u Department of Land Resources Management and Environmental Protection, Mekelle University, PO Box 231, Mekelle, Ethiopia; v International Platform for Dryland Research and Education, Tottori University, Tottori, 680-0001, Japan; w Universidad de la República, Facultad de Agronomía, Montevideo CP 12900, Uruguay; x UNISYSTEMS, Rue du Puits Romain 29 - Bertrange L-8070, Luxembourg; y Department of Science, Roma Tre University, Rome, Italy; z Department of Environmental Sciences, Environmental Geosciences, University of Basel, Basel, Switzerland

**Keywords:** Soil erosion, Hydrology, Risk, Soil health, Open data

## Abstract

Here, we present and release the Global Rainfall Erosivity Database (GloREDa), a multi-source platform containing rainfall erosivity values for almost 4000 stations globally. The database was compiled through a global collaboration between a network of researchers, meteorological services and environmental organisations from 65 countries. GloREDa is the first open access database of rainfall erosivity (R-factor) based on hourly and sub-hourly rainfall records at a global scale. This database is now stored and accessible for download in the long-term European Soil Data Centre (ESDAC) repository of the European Commission's Joint Research Centre. This will ensure the further development of the database with insertions of new records, maintenance of the data and provision of a helpdesk.

In addition to the annual erosivity data, this release also includes the mean monthly erosivity data for 94% of the GloREDa stations. Based on these mean monthly R-factor values, we predict the global monthly erosivity datasets at 1 km resolution using the ensemble machine learning approach (ML) as implemented in the mlr package for R. The produced monthly raster data (GeoTIFF format) may be useful for soil erosion prediction modelling, sediment distribution analysis, climate change predictions, flood, and natural disaster assessments and can be valuable inputs for Land and Earth Systems modelling.

Specifications Table

**Table:** 

Subject	Earth-Surface Processes; Hydrology and Water quality; Global and Planetary Change
Specific subject area	Rainfall erosivity dynamics and global rainfall intensity. Used as an input for soil erosion, sediment distribution, climate change, flood assessment and Earth systems models.
Type of data	Table with the data of annual rainfall erosivity and auxiliary information for 3939 stations. Table with the data of mean monthly erosivity. Shape file with all the stations and their erosivity values. 12 Raster (GeoTIFF) with global monthly erosivity at 1 km × 1 km resolution
How the data were acquired	At a global scale, this is the first time a data collection of observed (measured) high temporal resolution rainfall data (1 min, 5 min, 10 min, 15 min, 30 min, 60 min) took place. The collection of high temporal resolution rainfall data from the maximum possible number of countries was considered necessary to have a representative sample across climatic and geographic gradients.
	In the Universal Soil Loss Equation (USLE)-type soil erosion models [1], the rainfall erosivity parameter (R factor) describes the impact of rainfall on soil loss by water erosion. Rainfall erosivity accounts for the combined effect of rainfall duration, magnitude and intensity [2]. The calculation of erosivity is performed using high temporal scale rainfall data (30 min).
data format	Analysed and processed raw .xlsx format for the table data .shp format for the shape file .tiff format for the 12 raster files
Description of data collection	Primary rainfall data must be of a resolution of 60 min or less. The data should cover at least a period of 10-years (exceptions were done for areas where very few high temporal rainfall datasets exist). More than 100 data providers are listed in the acknowledgements and include Meteorological services, Environmental Institutions, Research organizations, Hydrological services and Academia (detailed list in the acknowledgments). The high temporal resolution rainfall data were processed according to the methodology described in Renard et al. [1] to calculate the rainfall erosivity. The results are given as mean monthly and annual records of rainfall erosivity (R-factor) per station.
Data source location	The presented data were processed, and they include a) point R-factor data from almost 4000 stations in 65 countries worldwide b) monthly erosivity values. 26 countries of the European Union (EU). 39 countries outside the European Union (EU): United Kingdom, Switzerland, Russian Federation (Europe), China, Japan, India, South Korea, Iran, Malaysia, Kuwait, Israel, Turkey (Asia and Middle East), the United States of America, Canada, Mexico (North America), Cuba, Colombia, Argentina, Brazil, Chile, Uruguay, Costa Rica, Jamaica, Suriname (South America & Caribbean), South Africa, Mauritius, Algeria, Democratic Republic of the Congo, Cape Verde, Cameroon, Eritrea, Ethiopia, Kenya, Niger, Nigeria, Rwanda, Tenerife, Zambia (Africa), Australia, New Zealand (Oceania).
Data accessibility	Repository name: Zenodo.org Data identification number: 10.5281/zenodo.8036998 Direct URL to data: https://zenodo.org/record/8036998
Related research article	Panagos, P., Borrelli, P., Meusburger, K., Yu, B., Klik, A., Lim, K.J., Yang, J.Y., Ni, J., Miao, C., Chattopadhyay, N., Sadeghi, S.H., Hazbavi, Z., Zabihi, M., Larionov, G.A., Krasnov, S.F., Garobets, A., Levi, Y., Erpul, G., Birkel, C., Hoyos, N., Naipal, V., Oliveira, P.T.S., Bonilla, C., Meddi, M., Nel, W., Dashti, H.A., Boni, M., Diodato, N., Van Oost, K., Nearing, M.A., Ballabio, C., 2017. Global rainfall erosivity assessment based onhigh-temporal resolution rainfall records. Sci. Rep. 7 (1), 4175. https://doi.org/10.1038/s41598–017–04282–8

## 1 Value of the Data

• The point data (annual and mean monthly R-factor) of GloREDa and the interpolated monthly erosivity datasets can be used for assessments in soil erosion, hydrology, sediment analysis, flood risk, natural hazards prevention and climate change;
• The point data of GloREDa can be used as reference data for scientists who study rainfall erosivity, soil erosion and future climate projections;
• The interpolated global monthly erosivity data can be used for modelling monthly global soil erosion and land degradation;
• The interpolated monthly erosivity data can be integrated in interdisciplinary modelling framework in Earth system models and ecosystem services communities.

## 2 Objective

Climate data records are particularly important for the scientific community and society. Although there are multiple global precipitation products, only a few of them account for heavy rainfall and extreme events. Rainfall erosivity is much different from precipitation as it includes the rainfall volume, duration, magnitude and intensity. Rainfall erosivity, known as the R-factor, is one of the input factors in the Universal Soil Loss Equation (USLE) prediction equation [1]. However, rainfall erosivity is mostly an approximation when it is estimated using only the rainfall volume (daily/monthly precipitation records) instead of the intensity. Quantifying the rainfall erosivity based on high temporal resolution (30 min) rainfall data is a very challenging task, mostly due to data scarcity [3]. We present the first ever global data collection of rainfall erosivity with almost 4000 stations covering 65 countries worldwide. The derived Global Rainfall Erosivity map [4] has been published in the Scientific Reports and is a success story with >3000 downloads from the European Soil Data Centre (ESDAC). As many users requested the original stations data, to develop customized analysis, spatial interpolation and modelling, we thought it appropriate to make the Global Rainfall Erosivity Database (GloREDa) available under the CC-BY license. In addition, we also calculated the monthly erosivity per station which allows for making better temporal soil erosion assessments.

## 3 Data Description

**Fig. 1:**
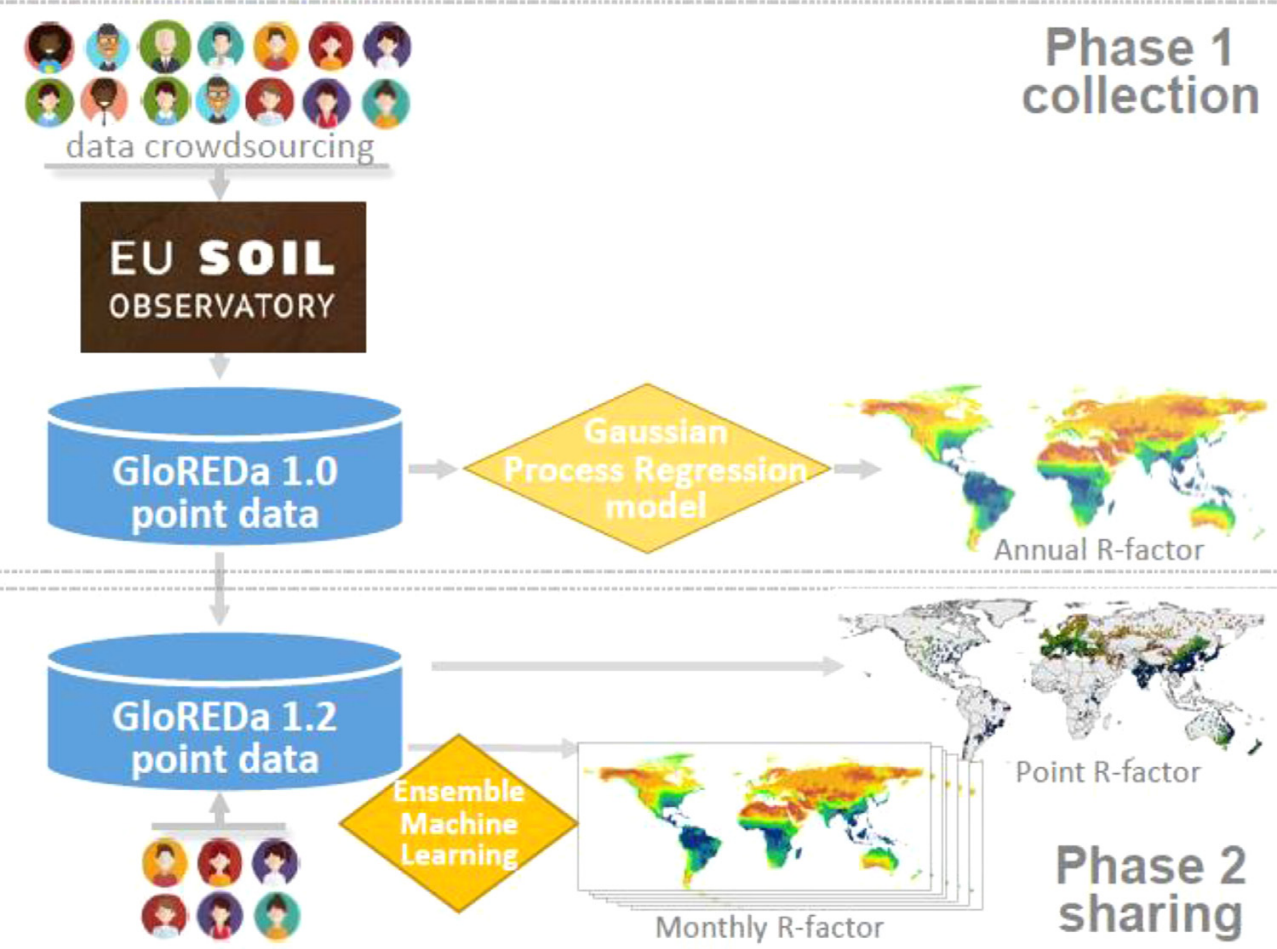
The development of Global Rainfall Erosivity Database (GloREDa) starting with phase 1 (2017) and the release of GloREDa 1.2 (2023).

The data collection started in 2013 with the objective to develop a pan-European assessment of soil erosion (Fig. 1 – phase 1). After the successful release of the pan European Rainfall Erosivity database and the derived R-factor map [5], we extended the data collection to a global scale. The first version of the Global Rainfall Erosivity Database (GloREDa) included data from 3625 stations distributed in 63 countries worldwide (Fig. 1). Based on GloREDa, we used a Gaussian Process Regression (GPR) to interpolate the erosivity values (R-factor) and to develop the first-ever Global Rainfall Erosivity map [4] which enabled a new present [6] and future global erosion assessments [7,8]. All produced maps were released in the European Soil Data Centre (ESDAC).

As the users who download the Global Rainfall Erosivity map have expressed the request to also have the station data from GloREDa, we are now releasing this database with this article (Fig. 1 – phase 2). In February 2023, the EU Soil Observatory (EUSO) working group on soil erosion made a call for a contribution towards the extension of GloREDa. In this call for data, contributors from Poland, Slovenia, Uruguay and the Democratic Republic of Congo (DRC) responded with data from 314 new stations. The contribution from DRC is very important, as the R-factor data are very limited for Africa [9]. Finally, the updated GloREDa 1.2 includes 3939 stations (Fig. 1).

In addition, we added the monthly component to GloREDa and we calculated the mean monthly R-factor per station. For 94% (3702 stations) of GloREDa, it was possible to add the monthly R-factor values summarizing 44,424 monthly records. This facilitated the development of the twelve global monthly R-factor maps (Fig. 1) similar to the European monthly erosivity maps [10].

Therefore, the current data release includes a) the annual R-factor for 3939 stations b) monthly erosivity values for 94% of the stations c) the Geographic Information System (GIS) shape file with the location of the GloREDa stations and d) the derived twelve (12) global monthly erosivity maps.

In the GloREDa database, we also provide ready to use auxiliary (meta) data which can assist those users interested in further spatial analysis, i.e., station name, exact co-ordinates, altitude, the period of measurement, the mean annual rainfall (provided by the data provider), the mean annual temperature and rainfall (extracted from WorldClim), the raw estimated R-factor, the temporal resolution of the input data and the final calculated R-factor. The data package includes amongst others the table with the R-factor data and the associated metadata that are described in the file “Explanation of field”.

The temporal scale is varying in GloREDa as the rainfall data have a resolution from 1 to 60 min. By applying scale factors tested in the literature [11], we converted all raw R-factor data to the 30-minutes erosivity (R_Final). This timescale was selected as an acceptable compromise between the coarse time resolution of 60 min and the higher ones (1–5 min). In addition, the 30-minutes erosivity time scale is the most used for the application of Renard et al. [1] equation.

**Fig. 2:**
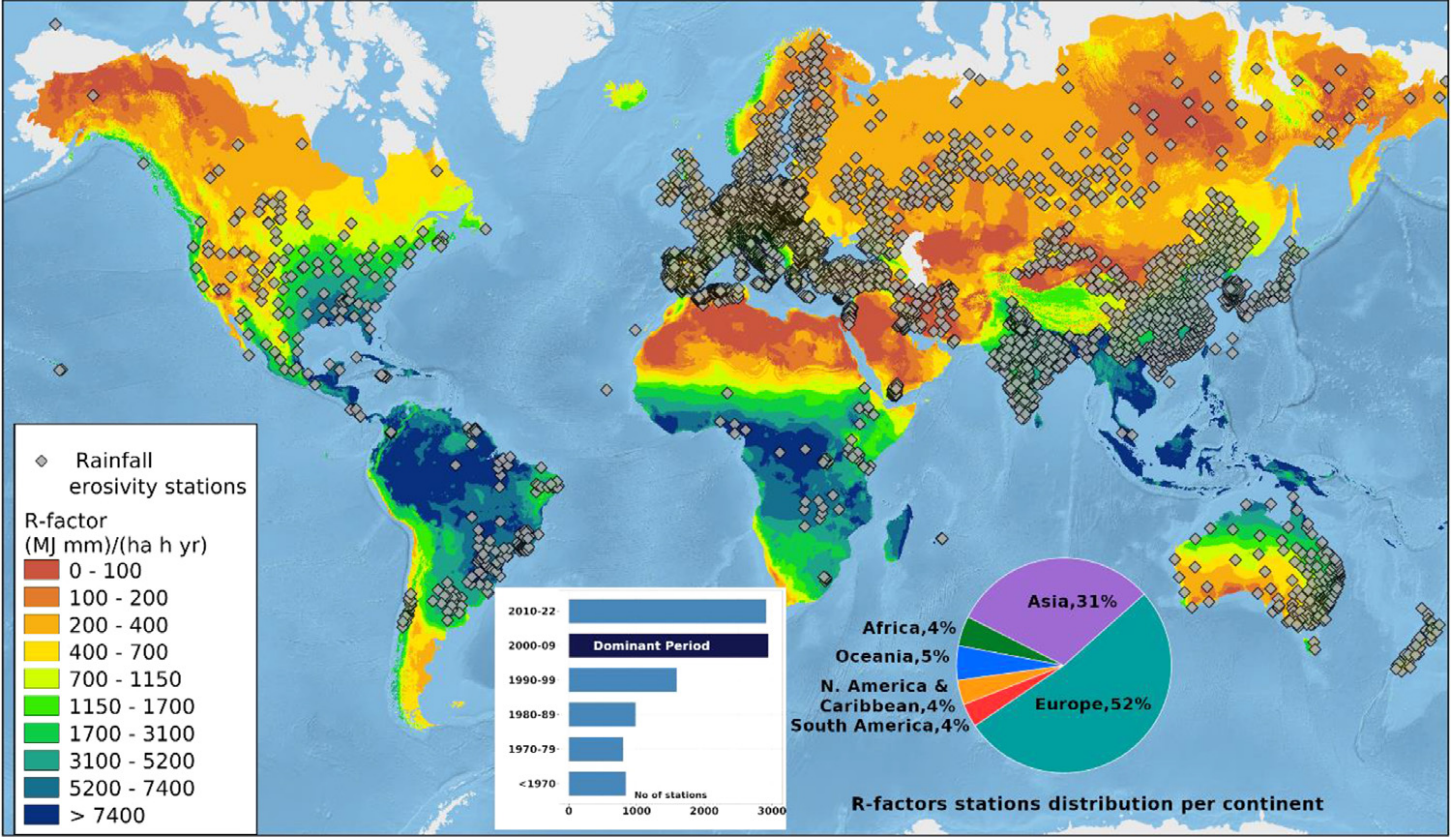
The distribution of the rainfall erosivity stations of GloREDa 1.2. Background map: The annual R-factor map.

Concerning the spatial distribution, 50% of GloREDa stations are in Europe (26 countries in the European Union plus Switzerland and the United Kingdom) because of established networks collecting high temporal scale rainfall data. Asia and the Middle East are well represented in GloREDa with 31% of the total stations distributed in ten countries (Fig. 2). The density of stations is relatively low (4%) in North America & Caribbean and in South America. Also in Africa, high temporal scale rainfall data are scarce and there is a lack of infrastructure to measure such data.

Almost 40% of the GloREDa stations is in the temperate climatic zone while 38.5% is in the continental zone. Of the total number of GloREDa stations, 15% is in the arid zone, while the tropics is under-represented (only 5.5%) and the polar zone has very few stations.

Currently, the dataset is covering 65 counties worldwide (Fig. 2). This is the first effort to gather and make available measured R-factor data across the globe. This collaborative effort initiated by the EU Soil Observatory (EUSO) will continue with future data collections that will seek to cover areas with low density of stations. Further contributions can be made to the database by downloading and completing the data according to template files available in the ESDAC data portal (https://esdac.jrc.ec.europa.eu/themes/global-rainfall-erosivity). Data submissions can be included in future data releases by contacting the listed data manager through the contact details listed in the ESDAC data portal.

**Fig. 3:**
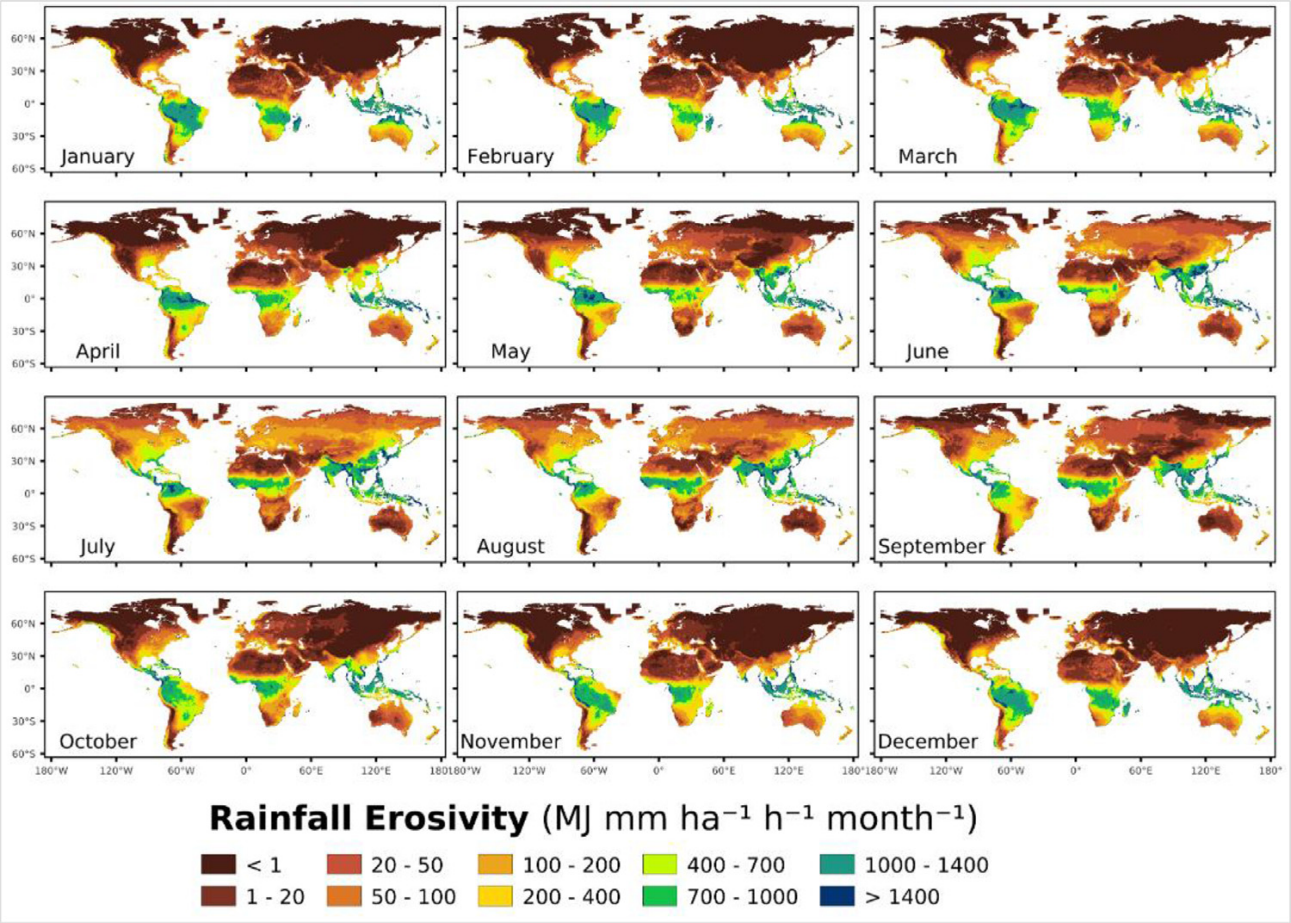
Global monthly rainfall erosivity datasets based on GloREDa.

We have also developed a global monthly rainfall erosivity datasets at 1 km spatial resolution (Fig. 3) using advanced machine learning models (as described in Material/Methods below). In absolute terms, July and August have the highest mean monthly R-factor (228 and 215 MJ mm ha^−1^ h^−1^ month^−1^) while February, April and November have the lowest means (around 152 MJ mm ha^−1^ h^−1^ month^−1^).

**Fig. 4:**
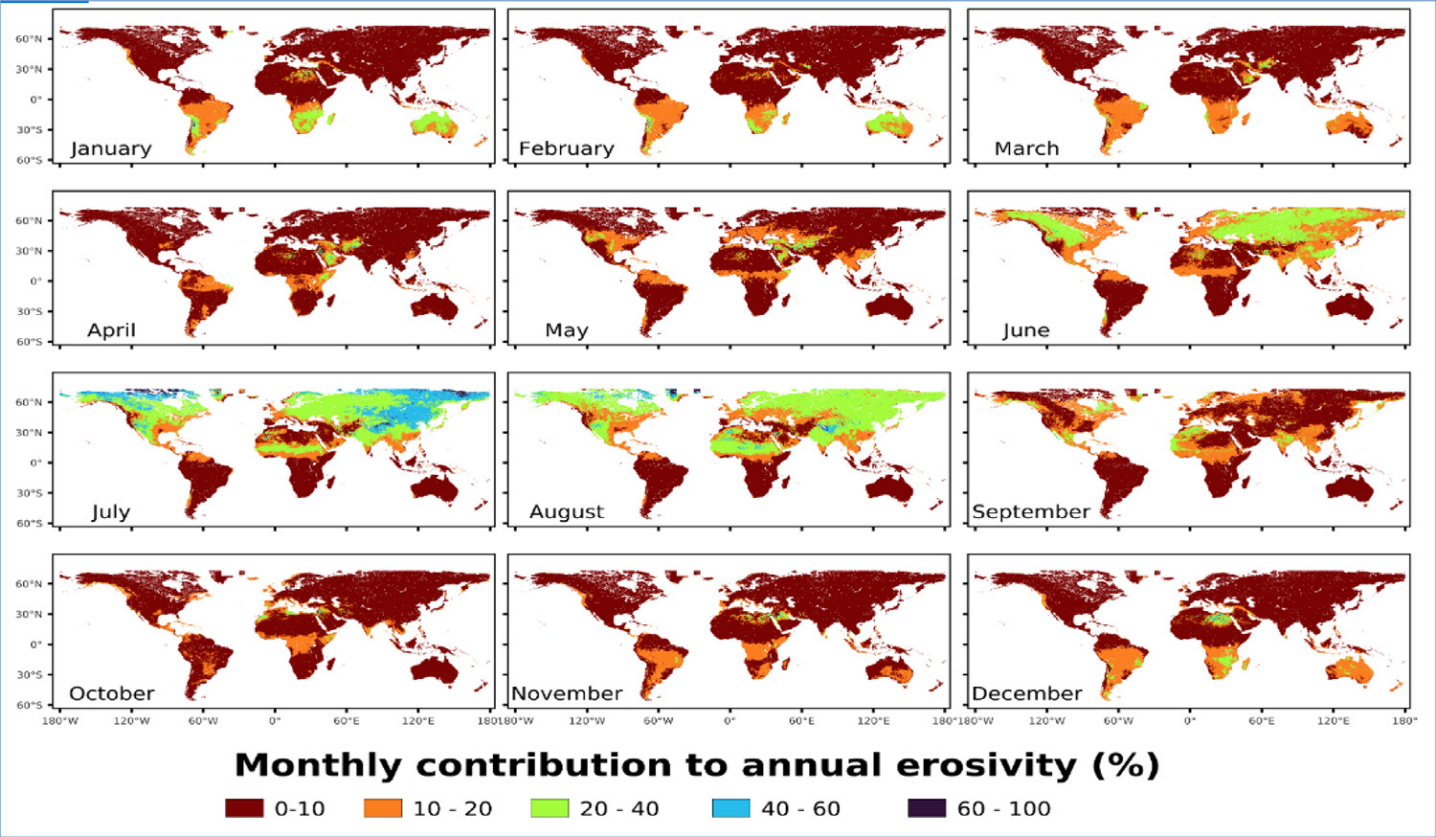
Contribution (%) of each month to the annual rainfall erosivity.

With the annual global mean R-factor of 2190 MJ mm ha^−1^ h^−1^ yr^−1^, there is a huge difference between the Northern and Southern hemispheres as the South has 3 times higher mean values compared to the North (4545 vs. 1545 MJ mm ha^−1^ h^−1^ yr^−1^). Summer period (June-July-August) for the Northern hemisphere contributes more than 47% of its total annual erosivity (Fig. 4). Winter months (December-January-February) contribute just 8% of the total annual erosivity in the North. In the Southern Hemisphere, the summer months (December-January-February) have a seasonal erosivity of 1848 MJ mm ha^−1^ h^−1^ which is 41% of the annual total erosivity (Fig. 4) while the winter season for the south (June-July-August) amounts to 458 MJ mm ha^−1^ h^−1^ (c.a 10% of the total).

Africa is the most balanced continent as the rainfall erosivity is well distributed during the whole year. The lowest mean monthly erosivity is in May and June (around 200 MJ mm ha^−1^ h^−1^ month^−1^) while September and August are the most erosive months (267 and 293 MJ mm ha^−1^ h^−1^ month^−1^). Oceania has its maximum erosivity in the season December to March (67% of the annual value). Asia has a significant concentration of rainfall erosivity from May to October (74% of the annual value). Europe and North America have similar monthly trends with picks in the summer season. South America has the highest annual erosivity amongst all continents with December to March experiencing extreme erosivity (circa 700 MJ mm ha^−1^ h^−1^ month^−1^) and the period July to August being also quite high (circa 300 MJ mm ha^−1^ h^−1^ month^−1^).

## 4 Data Limitations

The main limitation of the GloREDa is the different time periods covered by the calculated R-factor. However, 75% of the stations include data from the 2000 and onwards (Fig. 2 – dominant period). In addition, R-factor is a long-term average that takes into account annual records for at least 10 years. Short time series and different periods may cause a bias due to the temporal variation of R-factor. However, we are convinced that this risk due to the bias may be limited in a database with such a high number of records.

A second limitation is the lack of data in Africa and part of South America that is mainly due to lack of infrastructures for getting high temporal resolution rainfall records. Therefore, there is a room for improvement of the presented monthly erosivity maps with inclusion of more homogenous data inputs and data from missing parts of the world (Africa, South America, parts of Asia).

## 5 Experimental Design, Materials and Methods

The first part of this section includes the estimation of the rainfall erosivity (R-factor) from high temporal resolution rainfall data while the second part is on the model to interpolate the mean monthly erosivity values from the stations in order to develop high-resolution R-factor global monthly maps.

The compilation of GloREDa is based on aggregating the rainfall erosivity (R-factor) data per station. The calculation of rainfall erosivity is based on the original method [1] which first estimates the erosivity of a single rainfall event and then aggregates the calculated erosivity per year and month.

The calculation of rainfall erosivity (EI_30_) of a single rainfall event was based on the following equation:

(1) $$EI_{30}=\left(\sum_{r=1}^{k}e_{r}v_{r}\right)I_{30}$$

where e_r_ is the unit rainfall energy (MJ ha^−1^ mm^−1^); v_r_ the rainfall volume (mm) during the *r*th time period of a rainfall event divided in k-parts.

I_30_ is the maximum 30-minute rainfall intensity (mm h^−1^).

The unit rainfall energy (e_r_) is calculated for each time interval as follows:

(2) $$e_{r}=0.29\left[1-0.72e^{\left(-0.05i_{r}\right)}\right]$$

where i_r_ is the rainfall intensity during the time interval (mm h^−1^).

R-factor is the average annual rainfall erosivity (MJ mm ha^−1^ h^−1^ yr^−1^):

(3) $$R=\;\frac{\sum_{j=1}^{n}\sum_{k=1}^{m_{j}}{\left(EI_{30}\right)}_{k}}{n}$$

where

*n* is the number of years recorded,

*m_j_* is the number of erosive events during a given year *j* and

*k* is the index of a single event with its corresponding erosivity *EI_30_*.

The erosive rainfall events included in GloREDa comply with certain conditions of the RUSLE handbook [1] where a) the cumulative rainfall of an event is greater than 12.7 mm; (b) the event has at least one peak that is greater than 6.35 mm during a period of 15 min (or 12.7 mm during a period of 30 min) and, (c) where a rainfall accumulation of less than 1.27 mm during a period of six hours splits a longer storm period into two storms.

The calculation of the R-factor based on measured high temporal resolution rainfall data is an important advancement compared to past methodologies that have used empirical equations that estimate rainfall erosivity from monthly or annual rainfall totals. Unfortunately, many of these equations have been outdated and susceptible to misuse or applied erroneously to different places of the world [12].

Recently, high spatial and temporal resolution global precipitation estimates obtained by the National Oceanic and Atmospheric Administration (NOAA) or Integrated Multi-satellitE Retrievals for Global Precipitation Measurement (GPM-IMERG) are becoming more and more available. Such high spatial and temporal (30 min) resolution data have not yet been used for the estimation of rainfall erosivity on a global scale as they tend to smooth the high erosive events [13]. An alternative approach would be to merge the satellite-based precipitation products (NOAA, GPM-IMERG) with GloREDa in order to further improve the rainfall erosivity estimates [14].

We generated 1 km monthly rainfall erosivity maps using the Ensemble Machine Learning (EML) framework as implemented in the mlr package for R [15,[16], [17]. We overlaid the station point data with monthly covariates (global daily satellite rainfall data, CHELSA Climate Bioclimatic layers, NASA NEO long-term water vapour) and static information such as the Digital Terrain Model derivatives (elevation, slope, Topographic Wetness Index, terrain curvature). The DTM derivatives are at 250 m spatial resolution and originate from Global MERIT DEM. For example, point station data from May, was overlaid with MODIS land surface temperature, monthly precipitation, water vapour and similar from the corresponding month. Subsequently, we fit an ensemble model by using model-stacking with a meta-learner. As base-learners, we use Random Forest as implemented in the ranger package for R [18], XGboost: extreme gradient boosting [19], Cubist [20], and glmnet: GLM with Lasso or Elasticnet Regularization [21]. The results of the 5-fold Cross-Validation showed that the model achieved an R^2^ of 0.80 with best predictors being Satellite rainfall products (SM2RAIN), water vapour and MOD11A2 day-time temperature. The advantage of this approach, however, is that the SM2RAIN [22] and water vapour are atmospheric datasets produced independently of the stations used for training; hence, these data-streams are essentially independent and the mapping accuracy mentioned above is realistic.

## Ethics Statement

Not applicable

## CRediT Author Statement

**Panos Panagos:** Conceptualization, Methodology, Writing – original draft preparation, Writing – review & editing, Supervision; **Tomislav Hengl, Ichsani Wheeler:** Methodology, Software, Writing – original draft preparation, Validation; **Pawel Marcinkowski, Montfort Bagalwa Rukeza, Bofu Yu, Jae E. Yang, Chiyuan Miao, Nabansu Chattopadhyay, Seyed Hamidreza Sadeghi, Yoav Levi, Gunay Erpul, Christian Birkel, Natalia Hoyos, Paulo Tarso S. Oliveira, Carlos A. Bonilla, Mario Pérez-Bidegain, Werner Nel, Hassan Adl Dashti, Nejc Bezak, Kristof Van Oost, Saso Petan, Ayele Almaw Fenta** and **Nigussie Haregeweyn:** Data calculation, Reviewing; **Leonidas Liakos, Cristiano Ballabio:** Software, Data curation; **Pasquale Borrelli:** Visualization, Investigation, Conceptualization, Writing – review & editing.

## Declaration of Competing Interest

The authors declare that they have no known competing financial interests or personal relationships that could have appeared to influence the work reported in this paper.

## Data Availability

GloREDa (Original data) (Zenodo).

## References

[1] Renard K.G., Foster G.R., Weesies G.A., McCool D.K., Yoder D.C. (1997). Predicting soil erosion by water: a guide to conse.rvation planning with the revised universal soil loss equation (RUSLE). Agric. Handb..

[2] Nearing M.A., Yin S.Q., Borrelli P., Polyakov V.O. (2017). Rainfall erosivity: an historical review. Catena.

[3] Das S., Jain M.K., Gupta V. (2022). A step towards mapping rainfall erosivity for India using high-resolution GPM satellite rainfall products. Catena.

[4] Panagos P., Borrelli P., Meusburger K., Yu B., Klik A., Lim K.J., Yang J.Y., Ni J., Miao C., Chattopadhyay N., Sadeghi S.H., Hazbavi Z., Zabihi M., Larionov G.A., Krasnov S.F., Garobets A., Levi Y., Erpul G., Birkel C., Hoyos N., Naipal V., Oliveira P.T.S., Bonilla C., Meddi M., Nel W., Dashti H.A., Boni M., Diodato N., Van Oost K., Nearing M.A., Ballabio C. (2017). Global rainfall erosivity assessment based on high-temporal resolution rainfall records. Sci. Rep..

[5] Panagos P., Ballabio C., Borrelli P., Meusburger K., Klik A., Rousseva S., Tadić M.P., Michaelides S., Hrabalíková M., Olsen P., Aalto J. (2015). Rainfall erosivity in Europe. Sci. Total Environ..

[6] Borrelli P., Robinson D.A., Fleischer L.R., Lugato E., Ballabio C., Alewell C., Meusburger K., Modugno S., Schütt B., Ferro V., Bagarello V., Van Oost K., Montanarella L., Panagos P. (2017). An assessment of the global impact of 21st century land use change on soil erosion. Nat. Commun..

[7] Borrelli P., Robinson D.A., Panagos P., Lugato E., Yang J.E., Alewell C., Wuepper D., Montanarella L., Ballabio C. (2020). Land use and climate change impacts on global soil erosion by water (2015–2070). Proc. Natl. Acad. Sci..

[8] Panagos P., Borrelli P., Matthews F., Liakos L., Bezak N., Diodato N., Ballabio C. (2022). Global rainfall erosivity projections for 2050 and 2070. J. Hydrol. (Amst.).

[9] Bagalwa R.M., Chartin C., Baumgartner S., Mercier S., Syauswa M., Samba V.C., Zabona M.T., Karume K., Cizungu N.L., Barthel M., Doetterl S. (2021). Spatial and seasonal patterns of rainfall erosivity in the Lake Kivu region: insights from a meteorological observatory network. Prog. Phys. Geography Earth Environ..

[10] Ballabio C., Borrelli P., Spinoni J., Meusburger K., Michaelides S., Beguería S., Klik A., Petan S., Janeček M., Olsen P., Aalto J. (2017). Mapping monthly rainfall erosivity in Europe. Sci. Total Environ..

[11] Panagos P., Borrelli P., Spinoni J., Ballabio C., Meusburger K., Beguería S., Klik A., Michaelides S., Petan S., Hrabalíková M., Olsen P. (2016). Monthly rainfall erosivity: conversion factors for different time resolutions and regional assessments. Water.

[12] Chen W., Huang Y.C., Lebar K., Bezak N. (2023). A systematic review of the incorrect use of an empirical equation for the estimation of the rainfall erosivity around the globe. Earth-Sci. Rev..

[13] Bezak N., Borrelli P., Panagos P (2022). Exploring the possible role of satellite-based rainfall data in estimating inter- and intra-annual global rainfall erosivity. Hydrol. Earth Syst. Sci..

[14] Fenta A.A., Tsunekawa A., Haregeweyn N., Yasuda H., Tsubo M., Borrelli P., Kawai T., Belay A.S., Ebabu K., Berihun M.L., Sultan D. (2023). Improving satellite-based global rainfall erosivity estimates through merging with gauge data. J. Hydrol. (Amst.).

[15] Bischl B., Lang M., Kotthoff L., Schiffner J., Richter J., Studerus E., Casalicchio G., Jones Z.M. (2016). mlr: Machine learning in R. J. Mach. Learn. R..

[16] T. Hengl, L. Parente, C. Bonannella, (2022). Spatial and spatiotemporal interpolation/prediction using ensemble machine learning. Lecture notes (v0.1) OpenGeoHub foundation. Accessible at: https://opengeohub.github.io/spatial-prediction-eml/.

[17] Yamazaki D., Ikeshima D., Sosa J., Bates P.D., Allen G.H., Pavelsky T.M. (2019). MERIT Hydro: a high-resolution global hydrography map based on latest topography dataset. Water Resour. Res..

[18] M.N. Wright, A. Ziegler, 2015. ranger: A fast implementation of random forests for high dimensional data in C++ and R. arXiv preprint arXiv:1508.04409. 10.1093/bioinformatics/btq257 PMC2894507 20505004

[19] T. Chen, T. He, M. Benesty, V. Khotilovich, Y. Tang, H. Cho, K. Chen, R. Mitchell, I. Cano, T. Zhou, 2015. XGBoost: extreme gradient boosting. R package version 0.4-2, 1 (4), pp. 1–4.

[20] (1993). Combining instance-based and model-based learning. Proceedings of the Tenth International Conference on Machine Learning.

[21] Friedman J, Hastie T, Tibshirani R (2010). Regularization paths for generalized linear models via coordinate descent. J. Stat. Softw..

[22] Filippucci P., Brocca L., Massari C., Saltalippi C., Wagner W., Tarpanelli A. (2021). Toward a self-calibrated and independent SM2RAIN rainfall product. J. Hydrol. (Amst.).

